# Predictable Full Digital Workflow Using Stackable Surgical Templates for Complete Dental Arch Rehabilitation with Implant-Supported Fixed Restorations—Case Series and Proof of Concept

**DOI:** 10.3390/dj12110347

**Published:** 2024-10-30

**Authors:** Corina Marilena Cristache, Oana Elena Burlacu Vatamanu, Cristian Corneliu Butnarasu, Tamara Mihut, Eliza Denisa Sgiea

**Affiliations:** 1 Department of Dental Techniques, “Carol Davila” University of Medicine and Pharmacy, 8 Eroii Sanitari Blvd., 050474 Bucharest, Romania; 2 Doctoral School, “Carol Davila” University of Medicine and Pharmacy, 37 Dionisie Lupu Street, 020021 Bucharest, Romania; tamara.mihut@drd.umfcd.ro (T.M.); eliza-denisa.sgiea@drd.umfcd.ro (E.D.S.); 3 Megagen Dental Laboratory, 38 Delea Noua Street, 030925 Bucharest, Romania

**Keywords:** full digital workflow, stackable guides, virtual patient, edentulous maxilla, stackable surgical template, immediate fixed prosthesis

## Abstract

Background: In recent years, advancements in digital dentistry have provided new opportunities for more predictable and efficient treatment options, particularly in patients with failing dentition. This study aimed to evaluate the effectiveness and accuracy of a fully digital workflow using stackable surgical templates for complete dental arch rehabilitation with implant-supported fixed restorations. Methods: Four patients, comprising two males and two females with a mean age of 66 years, were included in this case series. Each patient underwent meticulous digital planning, including CBCT and intraoral scanning, to create a virtual patient for preoperative assessment and virtual treatment planning. The assessment of the trueness of implant positioning was conducted in Geomagic Control X software (version 2017.0.3) by referencing anatomical landmarks from both the preoperative and one-year postoperative CBCT scans. Results: A total of 25 dental implants were placed in the maxilla, followed by the installation of long-term provisional restorations. The results showed minimal deviation between the planned and actual implant positions, with mean 3D coronal, apical, and angular discrepancies of 0.87 mm, 2.04 mm, and 2.67°, respectively. All implants achieved successful osseointegration, and no failures were recorded, resulting in a 100% survival rate at the one-year follow-up. Patients reported high satisfaction with both the esthetic and functional outcomes based on their subjective feedback. Conclusions: The findings suggest that the use of a fully digital workflow with stackable surgical templates is a reliable and effective approach for immediate implant placement and prosthetic rehabilitation, enhancing treatment precision and patient comfort.

## 1. Introduction

Oral rehabilitation with dental implants has witnessed significant advancements over the past decades, particularly in the treatment of patients with terminal dentition [1]. Failing/terminal dentition, characterized by the end-stage of dental diseases leading to masticatory function loss, dentoalveolar alterations, teeth migrations, and esthetic impairment, poses a significant challenge in the field of dentistry. The primary obstacle in creating treatment plans for patients with failing dentition is the challenge of evaluating the proposed orientation of the occlusal plane, the positioning of the incisal edge, and the maxillomandibular relationship before implant surgery [2]. Digital advancements introduce valuable resources for the design and rehabilitation processes of these patients’ smiles, and masticatory function, incorporating the concept of a virtual dental patient [3]. These advancements include software programs available on the market that enable, in addition to surgical planning and prosthetic wax-up, the simulation of digital smile design, promoting better collaboration between patients, clinicians, and the interdisciplinary team through the use of a virtual dental patient model [3,4].

Despite the progress in dental implantology and restorative techniques, several challenges persist in achieving predictable outcomes that satisfy both esthetic and functional criteria. Most of the challenge comes from accurately transposing the planned implants into position in the patient’s oral cavity [5]. While digital planning offers precision and detailed guidance, the execution in a real-world setting introduces several variables that can compromise the intended outcome. Factors such as patient anatomy, bone density variations, surgical technique, and intraoperative adjustments can all impact the accurate transfer of the digital plan to the clinical setting. These hurdles emphasize the need for continuous refinement in both digital tools and surgical protocols to bridge the gap between planning and practice, ensuring optimal results for patients [6,7]. Traditional approaches often involve extensive treatment times, multiple procedures, and significant discomfort for the patient, alongside the challenge of achieving a harmonious integration with the patient’s dental and facial aesthetics [8].

In response to these challenges, the digitalization of dental processes has emerged as a pivotal evolution, offering greater precision, shorter treatment times, and improved patient outcomes. This approach leverages digital imaging, computer-aided design and manufacturing (CAD/CAM), and digital treatment planning to offer a streamlined, accurate, and minimally invasive treatment pathway. Among the most notable innovations is the development of a predictable full digital workflow using stackable surgical templates for complete arch rehabilitation with implant-supported fixed restorations [9,10].

The stackable guide protocol includes the following guides [11]:

- Base guide: fits in the vestibule of the dental arch and is stabilized with a minimum of three transverse pins (one anterior and two lateral) [8]. This base remains in place until the provisional prosthesis is fixed on the inserted implants;
- Tooth-supported surgical implant guide: used for implant placement when some of the remaining teeth are utilized for guide stabilization before extraction [10];
- Implant-supported guide: used for the insertion of all remaining implants after the extraction of teeth;
- Mucosal-supported guide: used for dental implant placement when the remaining teeth are few or absent;
- Bone reduction guide: if necessary, this guide is used for bone remodeling;
- Prosthetic guide: used for the positioning of the provisional fixed restoration.

Although numerous studies on the use of stackable guides have been published, most are limited to clinical case reports [12,13,14,15], with little focus on a comprehensive digital workflow that includes virtual patient creation. These existing reports often lack consistency in methodologies, making it difficult to assess the generalizability of their findings [8]. As the application of stackable guides continues to grow, there is a clear need for a standardized, repeatable digital protocol that can ensure implant placement accuracy across diverse clinical scenarios. The current literature remains scarce in describing a fully digital workflow that includes a thorough accuracy assessment of sequential stackable guides, underscoring the importance of establishing a more reliable and reproducible approach. Moreover, the criteria for determining the necessity of bone reduction in complete arch rehabilitation remain underexplored in the literature. The decision-making process for bone reduction, particularly in the context of immediate implant placement and provisional restoration fitting, plays a critical role in treatment outcomes. This unresolved issue is one that our study aims to address by providing a clearer framework for evaluating when and if bone reduction is necessary to achieve optimal restoration outcomes.

Therefore, the aim of this study was to describe a predictable, fully digital protocol for immediate implant placement and prosthetic rehabilitation of a complete maxillary dental arch, and to evaluate its accuracy across four consecutive cases, with particular emphasis on the role of the virtual dental patient in achieving successful clinical outcomes.

## 2. Materials and Methods

The study was conducted in accordance with ethical principles including the World Medical Association Declaration of Helsinki, the Belmont report, the Council for International Organizations of Medical Sciences (CIOMS) guidelines, and the International Conference on Harmonization in Good Clinical Practice (ICH-GCP). It received approval from the Ethics Committee of the ‘Carol Davila’ University of Medicine and Pharmacy (36368/2023, Bucharest, Romania). The research took place in a partner private clinic affiliated with the ‘Carol Davila’ University of Medicine and Pharmacy between January 2023 and June 2024. Four consecutive patients with failing maxillary dentition were enrolled in the present study [11]. A detailed presentation of one patient is in the Supplementary Materials.

The enrolled patients were subject to the following inclusion criteria: either experiencing failure of maxillary dentition or failing maxillary prosthetic restoration, necessitating and agreeing to full implants supported fixed rehabilitation; possessing good overall health with no contraindications for implant surgery; and exhibiting good mental health and the ability to fully comprehend and complete the consent form.

The exclusion criteria were: limited bone volume, requiring bone grafting; poor oral hygiene and lack of compliance; limited mount opening; and Parkinson’s disease.

### 2.1. Data Collection

The following data were mandatory for the creation of the virtual patient:

- Digital impression of the dental arches and occlusion registration in Centric Relation (CR) or intercuspal position (ICP) using Carestream 3600 (Carestream Dental LLC, Atlanta, GA, USA) intraoral surface scanner operated by DEXIS IS ScanFlow 1.0.10. (Envista Holdings Corporation Headquarters, Brea, CA, USA) and saved as STL (stereolithography) file format.
- CBCT, saved in Digital Imaging and Communications in Medicine (DICOM) format with a large field of view (FoV) 20 × 19 using NewTom™ VGi evo (NewTom Cefla S.C., Imola, Italy), performed in CR or ICP using a standardized protocol. Two scenarios were used to facilitate predictable file superimposing, depending on the number and position of the remaining teeth:

(a) for existing teeth, with no provisional removable prosthesis, three dental cotton rolls were placed in the buccal vestibule to move the lip away from the teeth, according to the “lip-lift” technique [16].
(b) for a reduced number of remaining teeth with an existing removable denture, radio-opaque composite spheres were placed on the existing denture to serve as markers.

- Facial scanning, performed with Bellus Arc 1 (Bellus3D Inc., Campbell, CA, USA) surface scanner, saved as OBJ (3D file format created by Wavefront Technologies, Paramount, CA, USA).
- One single operator performed intraoral scanning and facial scanning to ensure consistency.

### 2.2. Protocol for Virtual Dental Patient Creation

**Figure 1:**
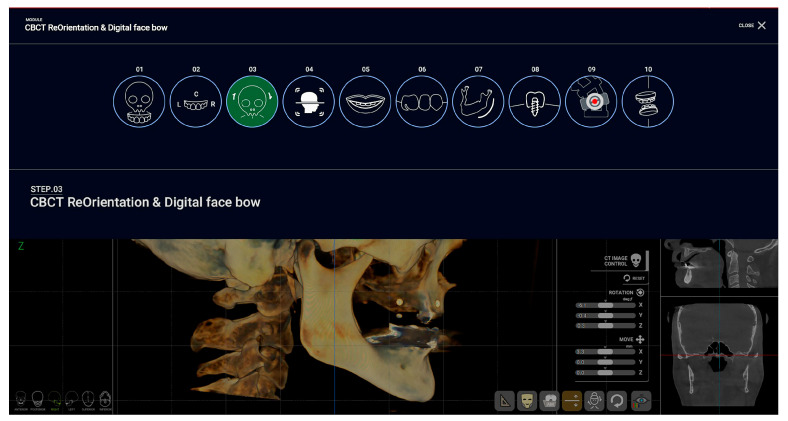
Overview of the steps involved in using R2Gate™ software for virtual patient creation and treatment planning.

For treatment planning and surgical template design, R2Gate™ software, version 2.0.0 (MegaGen, Daegu, Republic of Korea) was used. The software includes 10 features with corresponding steps: (1) CBCT and STL matching; (2) fine-tuning model matching and fine-tuning facial scan matching; (3) CBCT reorientation and digital facebow; (4) hard tissue cephalometric analysis; (5) 3D smile design; (6) wax-up design; (7) mandibular nerve tracing; (8) implant surgery; (9) quick guide and digital mounting; (10) adjusting the vertical dimension (VD) and Face Guide view [17] (Figure 1).

#### 2.2.1. Importing Data

All data collected are imported into a single folder with the patient’s name in the R2Data file. To ensure compatibility with the software, multiframe DICOM files were exported following the CBCT scan.

**Figure 2:**
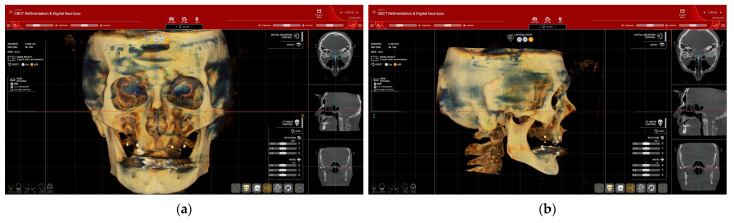
CBCT re-orientation (Patient #1): (**a**) frontal alignment of CBCT—centering and orienting with the orbital line parallel to the horizontal line; (**b**) lateral view with the Frankfort plane set as the horizontal plane.

Virtual patient creation starts with step no. 3, where CBCT is arranged to be centered and symmetrical and the Frankfort plane (drawn from Po—Porion to Or—Orbitale) is set as a horizontal plane (Figure 2).

#### 2.2.2. Merging Data

**Figure 3:**
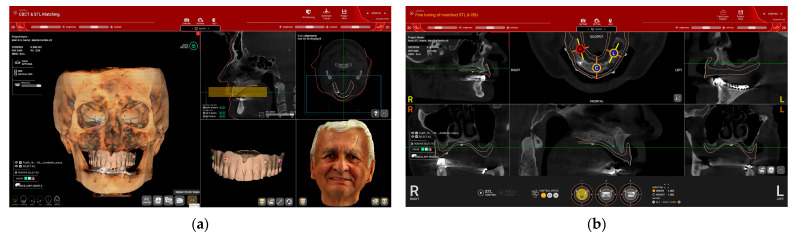
(Patient#1): (**a**) DICOM files from the CBCT, STL files from the intraoral scan (edentulous maxilla with preexisting adapted denture), and OBJ files from facial scanning were imported into the software; (**b**) fine manual alignment of the DICOM and STL files.

In steps 1 and 2, the intraoral (IOS) and facial scans are imported and matched with the CBCT file (Figure 3). The 3D alignment of the files, particularly between the CBCT and IOS, was completed in two stages: first, automatically by setting reference points (Figure 3a), and then manually for fine alignment (Figure 3b).

#### 2.2.3. Cephalometric Analysis

A cephalometric analysis is then performed (step no. 4) to evaluate the skeletal pattern of the patient for treatment planning and to verify the occlusal vertical dimension (OVD) and the preexisting occlusal plane direction.

**Figure 4:**
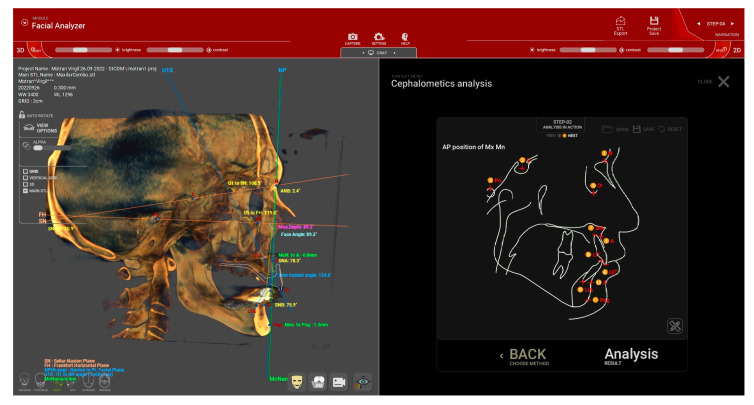
(Patient #1): Cephalometric analysis of the anteroposterior (AP) position of the maxilla (Mx) and mandible (Mn) for determining the A-Nasion-B (ANB) angle. Cephalometriclandmarks are displayed in the image on the right. N = nasion, S = sella, Or = orbitale, A = subspinale, UIA = upper incisor root apex, UIT = upper incisor incisal edge, LIT = lower incisor incisal edge, LIA = lower incisor root apex, B = supramentale, Pog = pogonion.

From the available options in the software, the AP (anteroposterior) position analysis of the maxilla and mandible (Mx Mn) can be selected to calculate the A-Nasion-B (ANB) angle. Before the measurement, several key points were marked on the 2D sagittal midsection of the CBCT: nasion (N), sella (S), orbitale (Or), subspinale (A), upper incisor root apex (UIA), upper incisor incisal edge (UIT), lower incisor incisal edge (LIT), lower incisor root apex (LIA), supramentale (B), and pogonion (Pog) (Figure 4). After these landmarks were set in the specified order, the software automatically calculated various angles. The sagittal jaw relationship was then classified based on the ANB angle: normal skeletal class I (0.3° to +4.8°), skeletal class II (>+4.8°), and skeletal class III (<0.3°) [18,19,20].

For edentulous patients, the reference dental points are marked based on the wax-up of the final restoration or the existing removable denture if it effectively restores the patient’s esthetic function. The position of the root apex is approximate, and as a result, the ANB angle value should be considered indicative rather than precise.

Based on the cephalometric analysis, the positions of point A and Pogonion (Pg) relative to the McNamara line are evaluated to assess the maxillary and mandibular positions in relation to the cranial base [21,22]. The McNamara line is defined as the perpendicular line drawn from the nasion to the Frankfort plane. In a normal maxilla, point A should lie on or slightly anterior (by approximately 1 mm) to the McNamara line. A negative value indicates a retruded maxilla, while a positive value indicates a protruded maxilla. Similarly, the analysis involving the position of point Pg relative to the McNamara line (normal range: −4 to 0 mm for females and −2 to +2 mm for males) interprets positive values as indicating a protruded mandible and negative values as indicating a retruded mandible [21,22]. The protrusion or retrusion of the maxilla and mandible, along with the occlusal vertical dimension and extent of bone atrophy, will guide the selection of the ideal type of restoration and the classification of the case according to the Prosthodontic Classification proposed by Misch (FP1, FP2, FP3, RP4, and RP5) [23].

**Figure 5:**
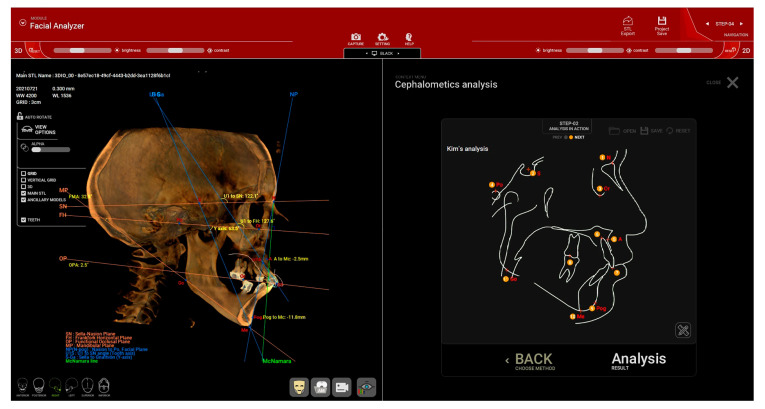
(Patient #2): Kim’s cephalometric analysis for assessing the positioning of the maxilla in relation to the McNamara line, as well as evaluating the Y-angle and occlusal plane (OP) angle for precise treatment planning. N = Nasion, S = Sella, Or = Orbitale, Po = Porion, A = Point A, 6 = the root apex of the upper incisor, 7 = the incisal edge of the upper incisor, 8 = the posterior point of occlusion, Pog = Pogonion, Me = Menton, Go = Gonion.

For the assessment of the appropriate vertical dimension and occlusal plane in patients with preexisting dentition or to verify the accuracy of provisional restorations, Kim’s analysis was employed. This analysis involves setting the following reference points: Nasion (N), Sella (S), Orbitale (Or), Porion (Po), Point A, the root apex of the upper incisor, the incisal edge of the upper incisor, the posterior point of occlusion, Pogonion (Pog), Menton (Me), and Gonion (Go) (Figure 5). The Y-angle, which is the angle between the Frankfort plane and the Sella–Menton (S-Me) line, serves as an indicator for the correct occlusal vertical dimension (OVD) setting. According to Down’s normative values for Caucasians, the average Y-angle is 59.4° (ranging from 53° to 66°) [24].

The occlusal plane angle (OP) (Figure 5) can be used to assess the appropriateness of the preexisting occlusal plane or to verify the direction of the newly established occlusal plane. Down’s analysis indicates that, for Caucasians, the occlusal plane angle should typically be around 14.5° (with a range of 3.5° to 20°) [24].

#### 2.2.4. Digital Facebow

**Figure 6:**
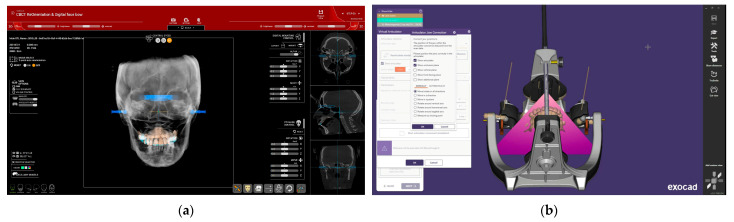
(Patient #2): Digital Mounting in the Articulator: (**a**) Artex articulator with the Frankfort horizontal plane selected as the reference plane in R2Gate™ software; (**b**) mounting imported as an STL file into EXOCAD software for the design of the provisional restoration.

In step #3 of the R2Gate software (CBCT reorientation and Digital Facebow), the center of the mandibular condyle is identified, and an appropriate type of articulator is selected from the available options. For all cases, the Artex articulator was chosen (Figure 6). The hinge axis of the selected articulator is then aligned with the hinge axis of the condyles. As shown in Figure 6a, the mounting plate of the articulator and the hinge axis are aligned with the maxillary model, and this configuration can be saved as an STL file for transfer to EXOCAD version 3.1 Rijeka (Exocad GmbH, Darmstadt, Germany) software. In EXOCAD, the hinge axis and mounting plate corresponding to those of the Artex articulator (Amann Girrbach AG, Mäder, Österreich) are used for the digital mounting of the models and for designing the prosthetic provisional (Figure 6b).

#### 2.2.5. Occlusal Plane Setting

**Figure 7:**
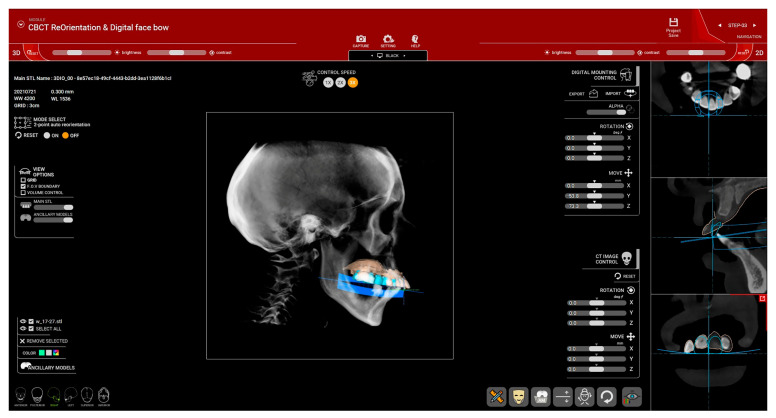
(Patient #2): Occlusal plane alignment using R2Gate™ software.

Additionally, in step #3 of the software, the central incisor edge is identified using the central cursor, and one of three available options for the digital occlusal plane is selected. These options correspond to different craniofacial types: 180 R for a wide (brachycephalic) type, 200 R for a normal (mesocephalic) type, and 220 R for a narrow (dolichocephalic) type (Figure 7). After importing, the occlusal plane can be adjusted, and the STL file is then exported to EXOCAD for provisional prosthesis design.

### 2.3. Prosthetically Driven Treatment Planning

#### 2.3.1. Design of the Provisional Restoration

**Figure 8:**
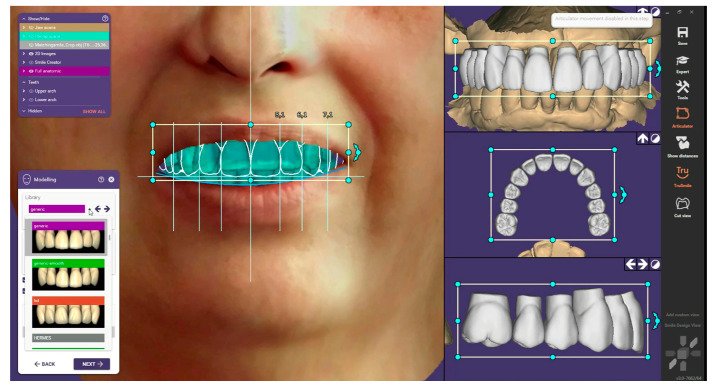
(Patient #2): Digital Smile Design in EXOCAD software for selecting anterior teeth, followed by designing the provisional restoration based on previously obtained information.

STL files of the maxilla and mandible IOS, OBJ file of the facial scan, virtual articulator mounting, and occlusal plane STL files were imported into EXOCAD software to design the provisional screw-retained prosthetic restoration (Figure 8).

#### 2.3.2. Implants Planning

**Figure 9:**
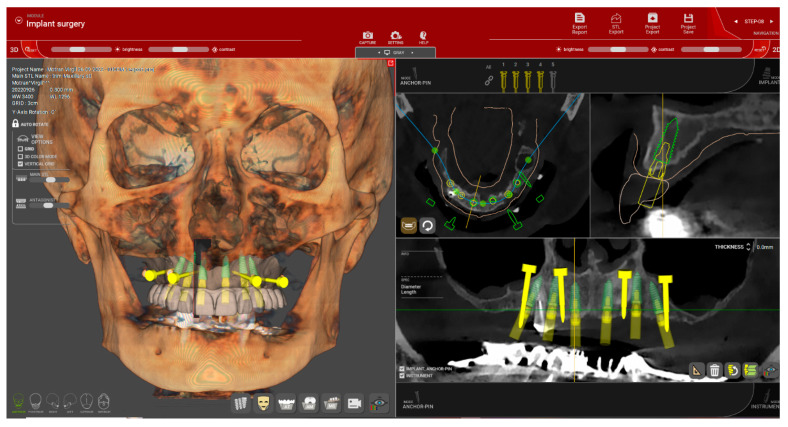
(Patient #1): Prosthetically driven implant planning. Six implants were planned for this patient, along with the design of four transverse pins for base guide stabilization.

**Figure 10:**
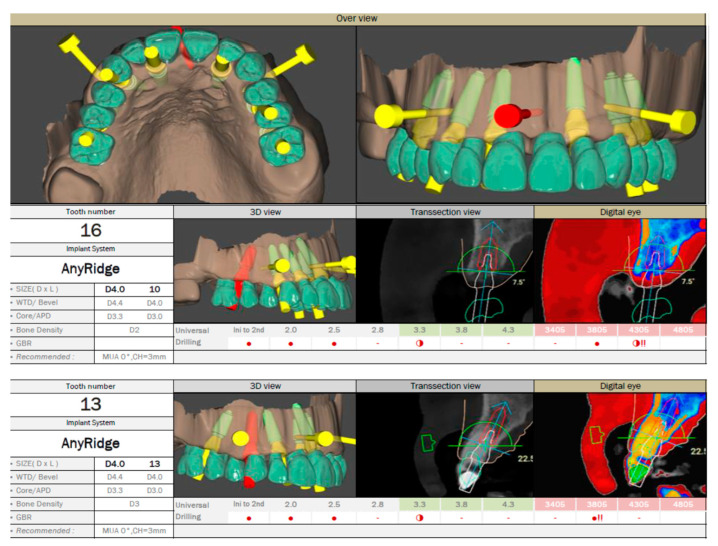
(Patient #2): Detailed positioning of the implants and transversal pins generated by R2Gate™ software according to the surgical plan. Bone density and the recommended drilling sequence for each implant position are also displayed. The R2Gate™ software converts the CBCT grayscale into five basic colors corresponding to the 256 shades of gray (right images): black represents air, red indicates soft tissue, blue represents soft bone, yellow corresponds to dense bone, and green represents high-density structures (such as enamel, cortical bone, and metal structures).

The STL file of the provisional restoration is imported into the R2Gate software, where the number, length, diameter, and position of the implants are planned according to the final restoration design. To facilitate bone quality assessment, the software’s “Digital Eye” option automatically converts the CBCT grayscale into five basic colors for preoperative bone density evaluation [5,25]. The implant planning data is then exported as an STL file for guide design. For patients with remaining teeth, a stepwise approach is planned for extraction and implant insertion, initially using a tooth-supported surgical guide. The software also provides a drilling sequence based on bone density and implant design. Figure 9 shows the treatment planning for a patient with a single remaining tooth, where one implant insertion guide was used. Figure 10 displays a drilling report for two of the six implants being inserted in a stepwise manner, with the first stackable implant guide supported by both mucosa and remaining teeth.

### 2.4. Stackable Guides Design and Manufacturing

**Figure 11:**
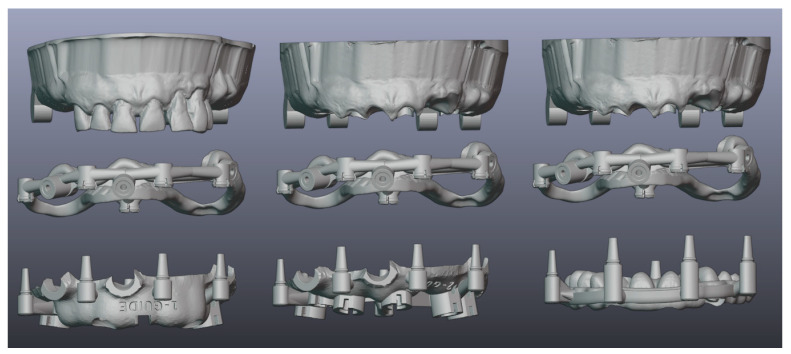
(Patient #3): Design of stackable guides (from left to right): maxillary model before extractions, base guide, tooth-supported guide for the insertion of the first two implants; model with virtual extractions, base guide, implant-supported guide; model with virtual extractions, base guide, and prosthetic guide.

Blenderfordental (B4D, Blenderfordental^®^ 2019) software was used to design stackable guides. The following guides were created for each patient: a base guide that fits into the vestibule of the dental arch, stabilized with three to four transversal pins, and a palatal pin if necessary, which remains in place until the provisional prosthesis is fixed on the inserted implants; a tooth-supported surgical guide for implant placement when some remaining teeth are used for stabilization before extraction; an implant-supported guide for the insertion of all remaining implants after the remaining teeth are extracted; and a prosthetic guide for the placement of the provisional fixed restoration (Figure 11).

**Figure 12:**
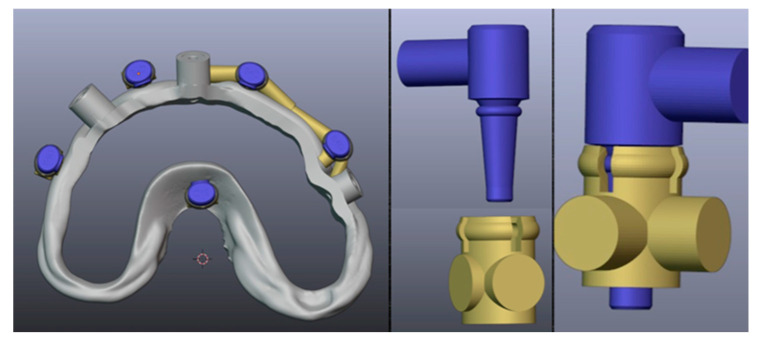
Matrix (displayed in blue)-patrix (displayed in beige) type of attachment between the base guide and subsequent surgical or prosthetic guides.

The connection between the base guide and the subsequent guides is established using specific attachments, as illustrated in Figure 12. These attachments ensure a secure and precise alignment of the guides during the surgical procedure, facilitating accurate placement of the implants.

**Figure 13:**
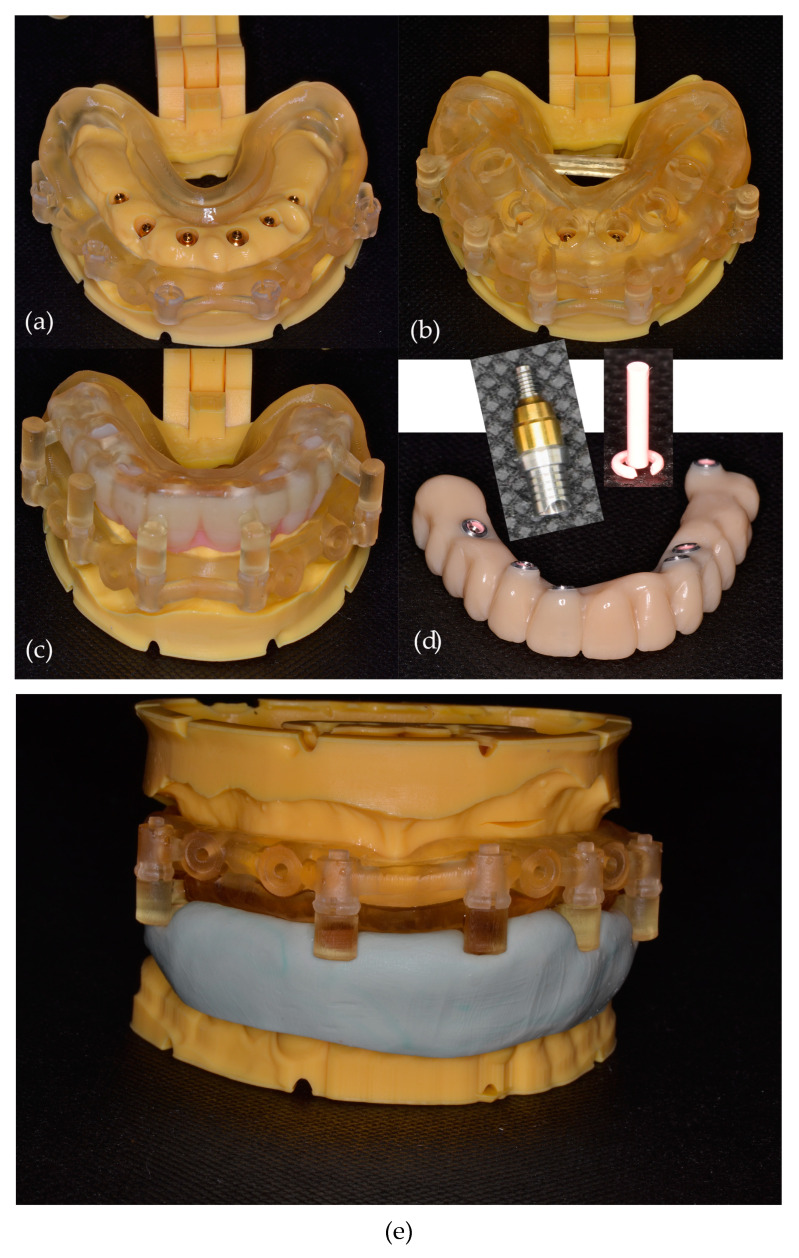
3D Printed Stackable Guides: (**a**) base guide on the 3D-printed model with implant digital analogs and OT Equator™ corresponding abutments in the planned positions; (**b**) mucosa-supported guide for dental implant insertion; (**c**) prosthetic guide with temporary fixed restoration; (**d**) temporary fixed restoration with temporary cylinders in place and Seeger™ conical Teflon rings (OT Equator™ abutments with temporary cylinders and Seeger™ conical Teflon rings are also displayed separately); (**e**) base guide and mucosa-supported guide with putty bite for base guide fixation using transverse pins.

The guides and models with virtual extractions were printed using the Phrozen Sonic XL 4 K printer (3Dream Teknoloji, Turkey), which employs Digital Light Processing (DLP) technology. Following post-processing, digital implant analogs were inserted into the models, and the corresponding abutments—OT Equator™ (Rhein83, Bologna, Italy)—along with temporary cylinders, adjusted in height to match the temporary restorations (Figure 13a–d).

For all patients, a long-term milled provisional restoration was fabricated using G-CAM material (Graphenano, Spain) (Figure 13d). In cases where patients had a reduced number of remaining teeth, a putty bite registration (Zeta Plus, Zhermack, Badia Polesine, Italy) was created to aid in the accurate positioning of the initial guide and the stabilization of the transversal pins (Figure 13e).

### 2.5. Implants Insertion and Provisional Restoration

The surgical procedures were conducted by an experienced surgeon (C.M.C.) in strict accordance with the manufacturer’s guidelines. The surgeries were performed under local anesthesia using a flapless and minimally invasive technique. Prior to implant insertion, a prophylactic antiseptic mouth rinse containing 0.2% Chlorhexidine (Corsodyl, GlaxoSmithKline, Brentford, UK) was administered for one minute to reduce bacterial contamination.

The base guide was correctly positioned and secured with transverse pins. In patients with remaining hopeless dentition, serial extractions were performed before placing the initial guide for dental implant insertion, following the preoperative plan.

For all four patients, AnyRidge implants (MegaGen, Daegu, Republic of Korea) were inserted according to the preoperative plan. The osteotomy sites were prepared based on preoperative bone density assessments obtained from CBCT scans and the drilling protocol provided by the planning software (Figure 10). The preparation was performed using shank-modified drills, which consist of three components: the stopper, the guide, and the drilling parts [4,5,25,26]. All implants were placed under fully guided conditions, and a hand ratchet was used to achieve the predetermined insertion depth at the designated anatomical landmarks.

OT Equator™ abutments, selected during the treatment planning phase based on the anticipated implant positions, were screwed onto the corresponding implants and torqued to 35 Ncm. These abutments, due to their geometry, can compensate for severe angulation discrepancies between implants (up to 80°, according to the manufacturer). The abutments were initially fitted onto the printed model (Figure 13a) to facilitate accurate matching during the insertion of the provisional restoration.

Temporary cylinders, adjusted for height, were mounted onto the OT Equator™ abutments. Using a prosthetic guide, the temporary fixed restoration was positioned and secured to the temporary cylinders with composite resin. The provisional restoration was then removed for final adjustments, including emergence profile contouring and gingival surface polishing. Seeger™ conical Teflon rings (Rhein83, Bologna, Italy) (Figure 13e) were placed between the prosthesis and the abutments to absorb functional shocks. After adjustments, the provisional restoration was screwed into place with a torque of 15 Ncm.

Following the placement, functionalization of the fixed provisional restoration was performed, and a panoramic X-ray was taken. Patients were provided with written postoperative care instructions, including the use of a 0.2% Chlorhexidine mouth rinse and a prophylactic antibiotic regimen of 1 g amoxicillin with clavulanate potassium, taken twice daily for the following seven days. Additionally, a nonsteroidal anti-inflammatory drug (Ibuprofen 400 mg) was prescribed for two days.

Patients were scheduled for follow-up appointments at one week, one month, six months, and one year postoperatively, with the long-term provisional restoration being replaced at the one-year mark.

### 2.6. Outcome Measurements

#### 2.6.1. One-Year Implants Survival Rate

Implant survival was assessed at each follow-up, up to one year, using the criteria proposed by Albrektsson et al. [27], which include the evaluation of mobility, radiographic peri-implant radiolucency, and the presence of symptoms such as pain, infection, or neuropathies, but without effectively measuring bone loss. Panoramic X-rays were performed at the one-week and six-month follow-ups to assess bone remodeling around implants.

A CBCT with a large field of view (FoV) 20×19, following the same protocol as at treatment planning was conducted at the one-year follow-up to assess the deviation between the planned and actual implant positions.

#### 2.6.2. One-Year Survival and Complications of the Provisional Fixed Prosthetic Restoration

At one week, one month, and six months, patients were recalled for clinical assessment, checking for proper tissue healing, assessment of bleeding, and inflammation.

All complications and appointments solicited by the patients were recorded.

Prosthesis success was determined based on function: if a prosthesis remained functional without requiring replacement, it was considered successful [28]. The incidence of mechanical complications, such as fractures of the fixed prosthesis and screw loosening, as well as biological complications, including soft tissue inflammation, fistula formation, or abscess development, was also assessed.

#### 2.6.3. Assessment of Trueness of Implants Insertion

**Figure 14:**
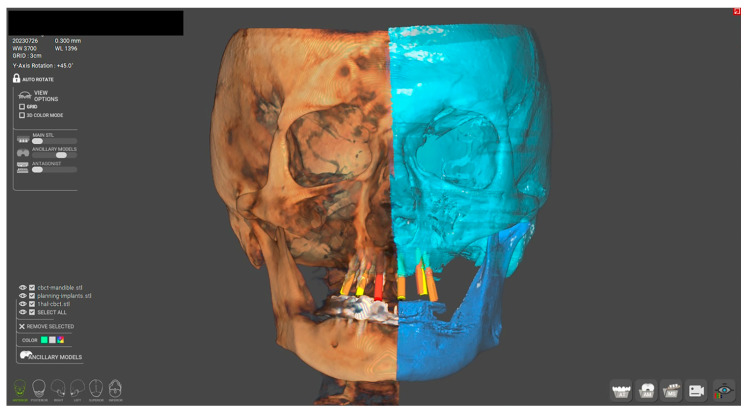
Aligned preoperative CBCT with treatment plan (right) and postoperative CBCT (left) in R2Gate software.

Assessment of trueness was performed by evaluating the deviation between planned and placed implants, expressed by three deviation parameters: 3D coronal, 3D apical, and angular according to a previously described technique [5,26]. The assessment of trueness in implant positioning was conducted by referencing anatomical landmarks from both the preoperative and one-year postoperative CBCT scans, which were performed using a strict protocol at the same radiologic center (FM Medident, Bucharest, Romania). The preoperative CBCT, along with the treatment plan saved as an STL file, was superimposed onto the postoperative CBCT in R2Gate software (Figure 14), using anatomical landmarks for alignment.

**Figure 15:**
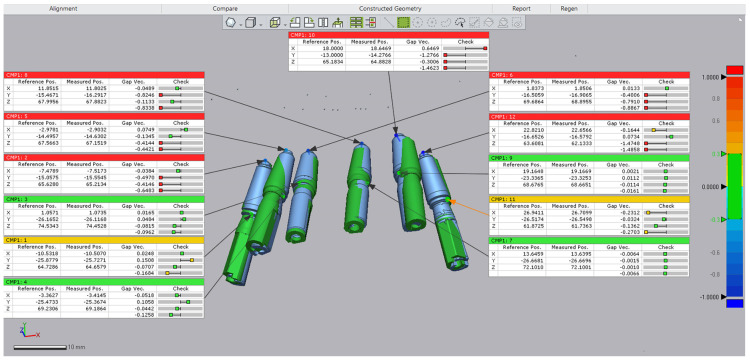
Assessment of the 3D positions of the planned implants (shown in blue) versus the placed implants (shown in green). Tolerance limits were set to ±0.1 mm (indicated by green on the scale). The 3D deviation for each point was calculated as the square root of the sum of the squared deviations along the three axes (x, y, and z).

In the postoperative CBCT, the corresponding implant library (with specific implant length and diameter) was applied, and the file was saved. Both the planned and actual implant position files were saved within the same coordinate system, and the assessment of trueness was performed in Geomagic Control X software, version 2017.0.3 (3D Systems, Rock Hill, SC, USA) with the STL of the planned implant position set as reference data (Figure 15).

## 3. Results

Four consecutive patients were included in this cohort study, comprising two males and two females, with a mean age of 66 years (standard deviation [SD] 7.87 years). According to the ANB angle measurements, two patients exhibited a normal skeletal class I profile (2.4° and 4.3°), while the other two patients were classified as skeletal class II (6.2° and 6.5°). Detailed patient characteristics are summarized in Table 1.

Except for patient #4, who exhibited a severe maxillary protrusion, the other three patients had measurements close to normal values. This was taken into consideration during provisional planning. The Y angle was within acceptable limits for all patients, indicating that the preset occlusal vertical dimension (OVD) was appropriate (Table 1).

Following meticulous digital planning, as previously described, a total of 25 AnyRidge implants were inserted into the maxilla. The implants varied in length from 8.5 to 13 mm, with a mean length of 11.38 mm (SD ± 1.29 mm), and in diameter from 3.5 to 4.5 mm, with a mean diameter of 4.08 mm (SD ± 0.24 mm) (Table 1). The position of the implants was determined based on the requirements of the prosthetic restoration, while the length and diameter of the implants were dictated by the available bone. No major complications were encountered during the insertion of the provisional restorations. All patients expressed satisfaction with the aesthetic outcomes, based on their subjective feedback, and necessary functional occlusal adjustments were performed during the adaptation session. Before the final tightening of the provisional restorations, adjustments were made to the mucosal aspect to facilitate the emergence profile conformation, followed by careful polishing to minimize the risk of mucosal irritation. Seeger™ conical Teflon rings were then positioned (Figure 13d, and the provisional restorations were secured over the OT Equator abutments with a torque of 15 Ncm. The occlusal access holes were subsequently sealed with Teflon tape and composite resin.

Further occlusal evaluations were conducted at one-week and one-month intervals. At the six-month follow-up, after conducting a panoramic X-ray assessment, the provisional restorations were removed to allow for clinical evaluation of gingival healing, with additional adjustments performed as needed. All patients completed the one-year follow-up period without any loss to follow-up. Successful osseointegration was observed in all implants, and no implant failures were recorded, resulting in a 100% survival rate. The long-term provisional restorations demonstrated satisfactory performance, with only two unscheduled appointments required by one patient for occlusal adjustments within the first six months.

Implant deviations from the planned positions, measured as mean (SD) for 3D coronal, apical, and angular discrepancies, were 0.87 (±0.50) mm, 2.04 (±0.69) mm, and 2.67° (±1.25°), respectively. At the one-year follow-up, the long-term provisional fixed restorations were replaced with definitive zirconia-on-titanium milled restorations.

## 4. Discussion

The present study aimed to establish a protocol for the full fixed rehabilitation of a maxillary dental arch with failing dentition. There were two main reasons for choosing a fully digital workflow for restoring the maxilla: first, intraoral scanning is more predictable for the maxilla due to the fixed palatal mucosa, whereas the mandible has less stable mucosa on the residual ridge, making digital impressions less accurate. Second, maxillary restoration is crucial for achieving esthetic outcomes.

The protocol consists of several steps: initially, the creation of a virtual dental patient, followed by prosthetic design and implant planning. Subsequent steps include the design and production of surgical guides and the manufacturing of the provisional prosthesis. By following this protocol, both surgical and prosthetic restoration become more predictable, enabling immediate esthetic restoration. This standardized, fully digital workflow not only enhances precision in implant placement but also minimizes variations between planned and actual outcomes. The collaborative approach involving the entire clinical team, alongside the use of virtual simulations, ensures that treatment is tailored to each patient while maintaining consistency and predictability across cases.

For the first guide design, the number of pins is carefully determined based on the available bone, remnant dentition, and bone density, which we assess using the “Digital Eye” feature in the R2Gate software. This allows for precise evaluation of the necessary number of pins, as more pins do not always equate to better stabilization. In fact, in cases of dense mandibular bone, exceeding three pins can create internal tensions that may lead to guide fracture. Four transverse pins were used for each of the four enrolled patients (Figure 9 and Figure 13e), in line with other published case reports [12,15,29,30].

Another important consideration is the manufacturing technique and material used for the first guide. We employ DLP (Digital Light Processing) technology and use a dedicated resin (SG Surgical Guide) from NexDent (Vertex-Dental B.V., Centurionbaan, the Netherlands), ensuring a minimum thickness of 3 mm. This combination provides the guide with excellent mechanical properties, allowing for durability and stability during the surgical procedure [31]. In some clinical reports, guides were manufactured using selective laser melting (SLM) from cobalt–chromium alloy powder [32]. We preferred the use of NextDent resin and the DLP technique for several reasons. First, the guides were mucosa-supported (or mixed teeth and mucosa), and the maxillary mucosa had a certain degree of resilience, which could be compensated for by the low resilience of the resin. Additionally, the use of resin guides presents a more cost-effective option. Moreover, due to the large dimensions of the first guide, not all CAM centers had the capability to sinter the guide, limiting its accessibility.

The stackable components of the guides are connected using various types of attachments, such as magnets [13,33], balls [29], screws [9,12,30], or notches [34], depending on the preference of the medical team. To date, no study has proven one system to be more efficient than another. In our study, we used a matrix-patrix system designed in Blender4Dental with an adjustable offset (Figure 14), depending on the required retention between the guides.

While several case reports and case series have been published on the use of stackable surgical guides, most of these studies involved open-flap surgery and the use of a bone reduction guide to establish a suitable ridge for implant placement and to accommodate a bone-supported drill guide [8,35,36,37].

This protocol introduces several innovations compared to previously published case reports and series [12,13,38]. One significant innovation is the creation of a virtual dental patient and the clinically oriented cephalometric analysis performed to gather information for planning the provisional restoration. The introduction of the virtual dental patient significantly improves upon previous methods by allowing enhanced preoperative planning, visualization, and communication between the clinical team and the patient. This digital approach reduces treatment time by enabling more precise implant positioning and prosthetic design, minimizing the need for adjustments during surgery.

Another novel aspect is the use of OT Equator abutments as part of the OT Bridge protocol to compensate for the angulation between the inserted implants and the requirements for restoration. In addition to compensating for implant angulation, these abutments facilitate bone remodeling. Their design eliminates the need for space to accommodate angulation correction components, such as multi-unit abutments (MUAs), thereby reducing the necessity for a bone-reduction stackable drill guide. The OT Equator abutment offers several advantages, such as a short profile, narrow emergence, and the ability to resolve implant divergence while reducing tensions in the structure due to the Seeger solution. Moreover, the Seeger ring system helps manage higher stresses associated with a single-piece maxillary fixed prosthetic reconstruction during functional loading [39].

The use of a long-term milled provisional material, G-CAM (Graphenano, Spain), allows the temporary fixed restoration to be maintained for up to one year, enabling adjustments and bone remodeling due to its favorable properties. The graphene nano-reinforced biopolymer G-CAM disc is specifically designed for permanent dental structures and is available in different shades to provide a natural aesthetic appearance. It offers superior mechanical, physicochemical, and biological properties compared to other materials currently used in the sector [40]. In comparison to unmodified Poly(methyl methacrylate) (PMMA), G-CAM, which incorporates graphene-like materials, offers improved properties such as enhanced flexural strength and fracture toughness. According to the study by Agarwalla et al. [41], while G-CAM showed similar translucency and hardness to unmodified PMMA, it demonstrated mechanical strength and reliability that make it suitable for CAD/CAM prosthetic restorations, including complete arch restorations. Furthermore, the use of graphene-like materials improves the performance of PMMA-based resins, allowing them to withstand higher occlusal forces, which is a significant advantage over traditional PMMA. The robust mechanical properties of G-CAM eliminate the need for metal-reinforced PMMA structures [42], thereby reducing the risk of fracture during the healing period.

Key factors in the dental rehabilitation procedure include the hinge axis position, centric relation (CR) or intercuspal position (ICP), and vertical dimension of occlusion (VDO) [43]. This information is obtained during the virtual patient creation and analysis. Additionally, the maxillary position relative to the hinge axis was transferred as a virtual articulator mounting (Artex for all four patients) into the software for provisional prosthesis design, eliminating the need for a conventional facebow and model mounting.

For implant position planning, provisional design, and stackable guide design, three different software programs were utilized: R2Gate, EXOCAD, and Blender4Dental. R2Gate was used for virtual patient creation and analysis, operating on a pay-per-export basis. EXOCAD, a widely recognized software in dental laboratories, was employed for the design of provisional restorations under a paid license. Blender4Dental, a recently introduced and cost-effective paid software, was used for designing the surgical guides.

The mean deviation between the planned and actual implant positions was 0.87 mm (±0.50 mm) at the coronal level and 2.04 mm (±0.69 mm) at the apical level. These values are comparable to those reported in other studies, although slightly lower at the apical level, which ranged from 0.44 mm to 1.43 mm for coronal deviations and 0.887 mm to 1.90 mm for apical deviations [32,33,37,44]. The observed higher apical deviation in this study can be attributed to the implant length and the softer maxillary bone, compared to other studies that primarily assessed the mandible. The mean angular deviation was 2.67° (±1.25°), which is consistent with other studies reporting mean values between 2.4° and 4.14° [32,33,37,44]. Overall, all deviations observed were within acceptable clinical limits [45].

A limitation of this study is the small sample size, consisting of only four participants (two males and two females), and the lack of a comparative group using guided implant insertion without stackable guides. However, the authors’ previous experience with immediate loading of the full maxilla without using prosthetic stackable guides resulted in suboptimal outcomes, highlighting the potential benefits of the approach evaluated in this study.

Another challenge is the inherent learning curve associated with adopting digital workflows and stackable guide systems, particularly for clinicians unfamiliar with these technologies. This learning curve may influence the outcomes and efficiency of the treatment process.

The fully digital workflow and the use of stackable guides allowed for more personalized treatment by providing precise control over implant positioning and prosthetic design, tailored to each patient’s anatomical conditions. The ability to visualize and simulate treatment outcomes using virtual planning helped ensure that the restorations were both functional and aesthetically aligned with the patient’s needs.

A Randomized Clinical Trial (RCT) study comparing the stackable guide protocol and the conventional All-on-6 protocol in the maxilla could provide valuable insights into the benefits and limitations of each approach. Additionally, further studies with larger sample sizes and comparative groups would help assess the broader efficacy of this method and refine the protocol for more complex anatomical situations. This would provide a more comprehensive understanding of how the fully digital workflow and stackable guide system perform in different clinical scenarios.

## 5. Conclusions

This report on four consecutive clinical cases of failing maxillary dentition highlights the effectiveness of a fully digital workflow using stackable surgical templates for the immediate rehabilitation of a complete maxillary arch. By leveraging virtual patient creation, precise implant placement, and innovative materials, this approach enhances treatment predictability and improves patient outcomes, setting a benchmark for future advancements in digital dentistry.

## Figures and Tables

**Table 1:** Patient characteristics and distribution of inserted implants.

	Patient #1	Patient #2	Patient #3	Patient #4
Gender (M/F)	M	F	F	M
Age	75	58	61	70
Number of remaining hopeless teeth (maxilla)	1	9	6	7
ANB angle (°)	2.4	6.5	6.2	4.3
McNamara line to A (mm)	2.2	−2.5	−0.6	12.3
Y angle (°)	57.8	63.5	59.5	53.7
Implants inserted nr./diameter (mm) × length (mm)	6/4 × 11.5	1/4 × 10; 2/4 × 13; 1/3.5 × 13; 1/4.5 × 11.5; 1/4.5 × 10	1/4 × 11.5; 2/4 × 13; 1/4.5 × 8.5; 2/4 × 10	2/4 × 13; 3/4 × 10; 2/4.5 × 11.5

## Data Availability

Further data are available upon request from the corresponding authors.

## Supplementary Materials

The following supporting information (Case Presentation: Patient #1) can be downloaded at: https://www.mdpi.com/article/10.3390/dj12110347/s1.
