# Supplementary material for: Predictable Full Digital Workflow Using Stackable Surgical Templates for Complete Dental Arch Rehabilitation with Implant-Supported Fixed Restorations—Case Series and Proof of Concept
Source: Dent J (Basel). 2024 Oct 30;12(11):347. doi: 10.3390/dj12110347 (PMC11593087; doi:10.3390/dj12110347)
Supplement: Supplementary file 1 [file dentistry-12-00347-s001.zip › dentistry-3205578-supplementary.pdf]

Case Presentation: Patient #1

A 75-year-old male with partially edentulous maxilla (#13 remaining), presented with a failing maxillary denture that had been fractured and repaired multiple times. The patient sought an implant-supported fixed restoration.

a 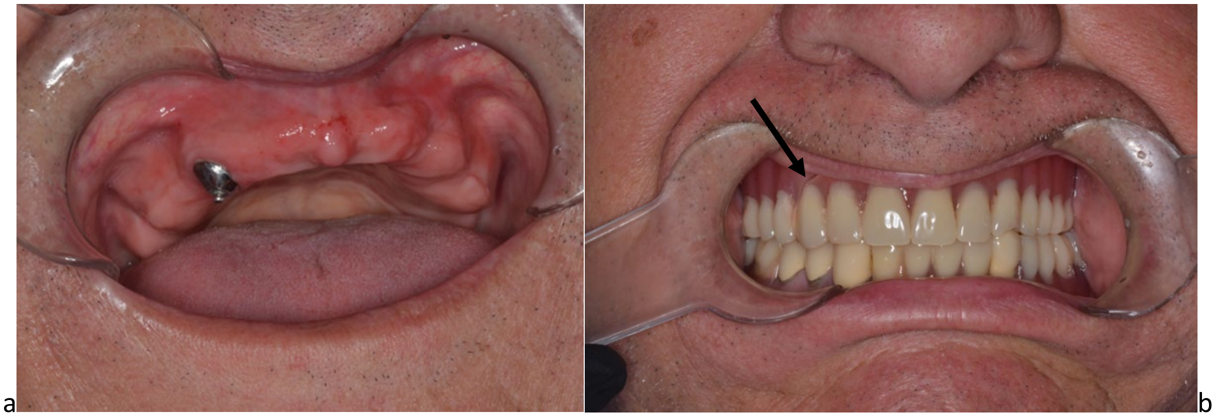 b

a)Intraoral view of the partially edentulous maxilla; b) Old denture in place. The arrow shows denture's fracture line.

Preoperative Assessment:

To facilitate accurate superimposition, radio-opaque composite spheres were placed on the existing denture to serve as markers. Intraoral scans were performed with the denture in place, and the internal surface of the denture was also scanned to assist in the superimposing process.

Treatment Planning:

The process of creating a virtual patient was detailed in the manuscript. A fully digital workflow was used to plan the provisional restoration design and the positioning of the implants. The digital treatment plan included the following steps:

- **Implant Positioning:** Precise implant positions were planned digitally based on the virtual patient.

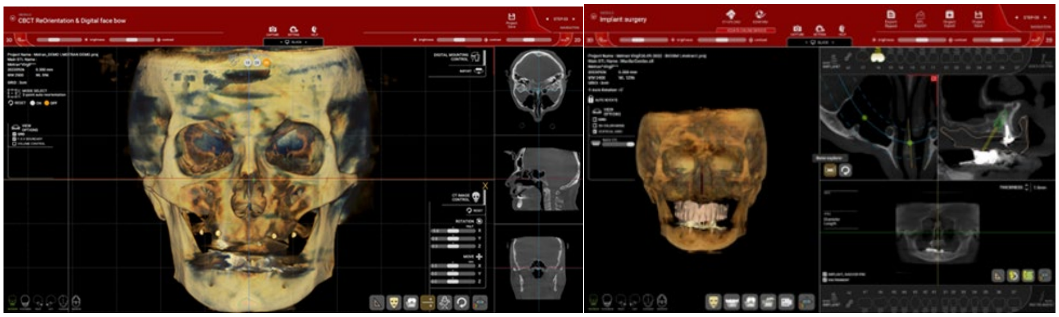

Virtual patient creation in R2Gate software

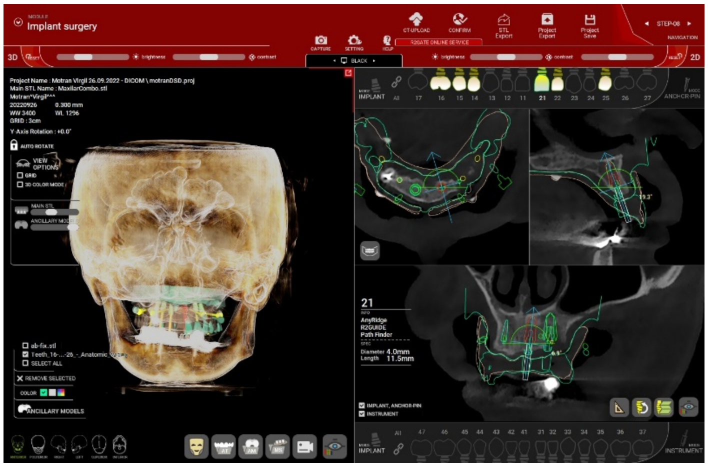

Implants position planning

Stackable Guides:

- *Guide 1*: The first guide was designed for accurate placement of the OT Equator abutments on the digital implant analogs.
- *Guide 2*: The second guide was used for dental implant insertion.
- *Guide 3*: The prosthetic guide was used for the provisional restoration.

a 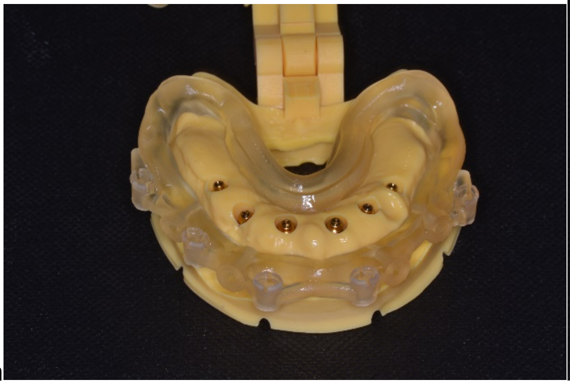 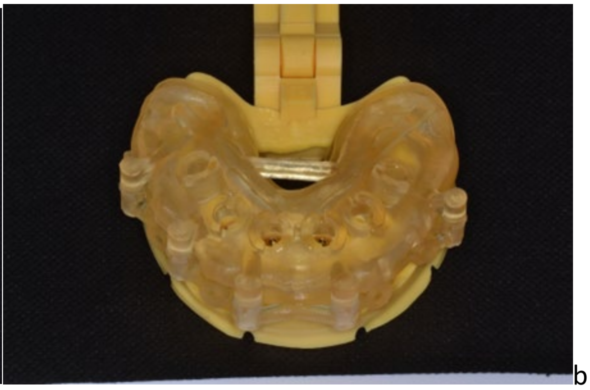 b

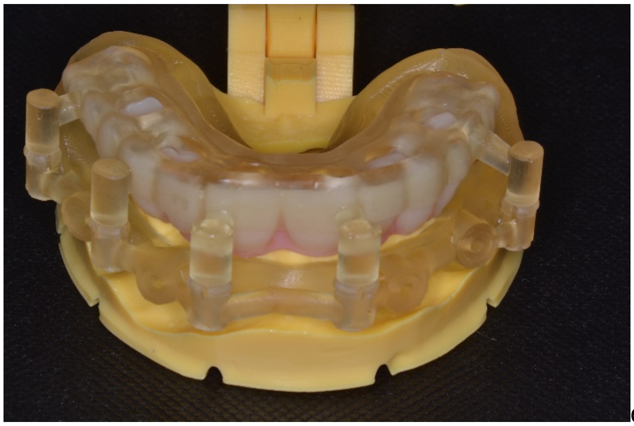 c

a. First guide on the model with the corresponding OT Equator abutments inserted on the digital implants’ analogues.
b. Second guide for dental implants insertion
c. Prosthetic guide with provisional

Surgical Procedure and Provisional Restoration:

During surgery, the first guide was fixed using transverse pins. Implants beds were prepared, and OT Equator abutments were placed over the implants. Temporary cylinders, adjusted to the required length, were inserted over the OT Equator abutments.

The provisional restoration was then successfully placed.

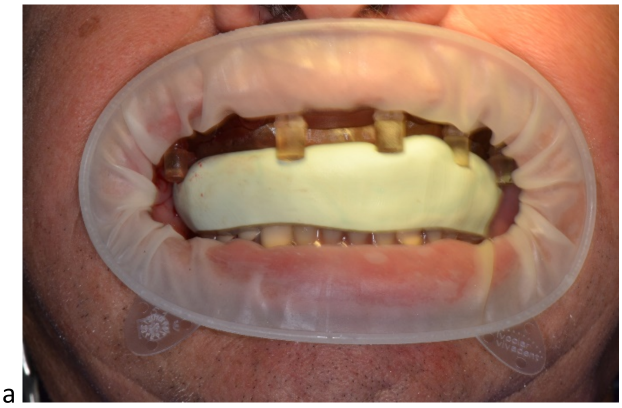
a

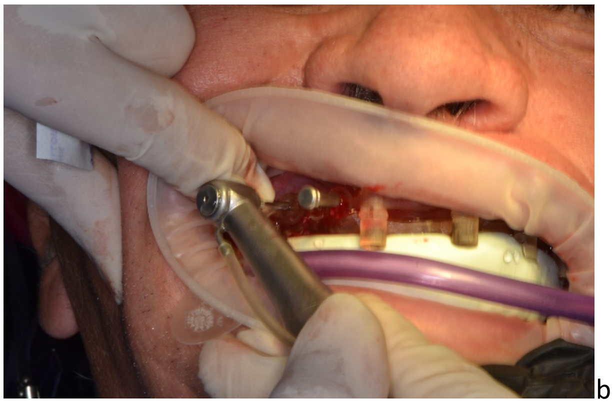
b

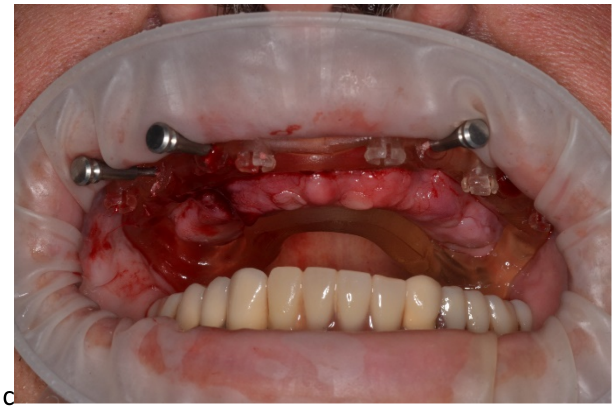
c

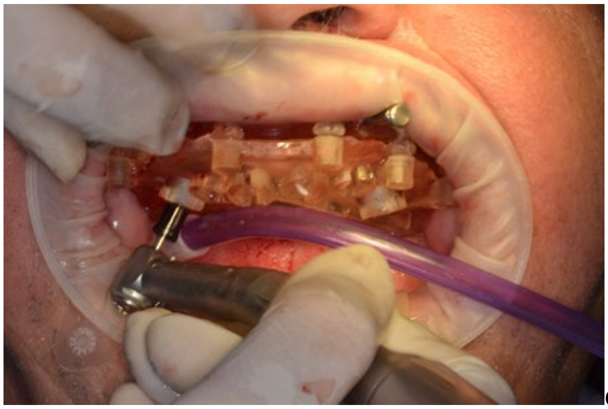
d

e 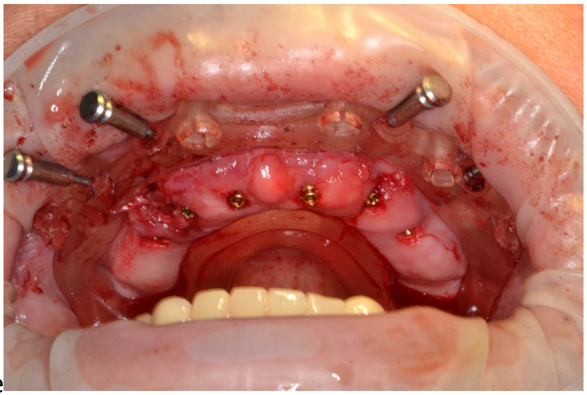 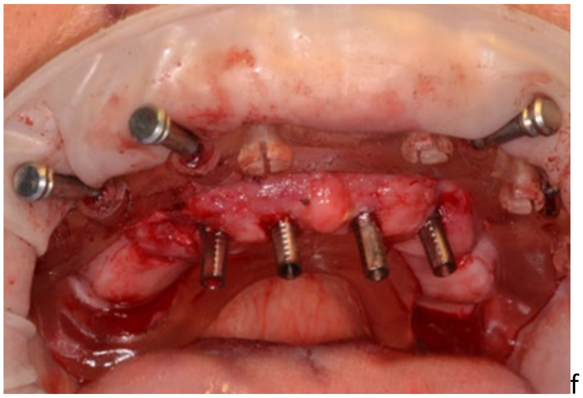 f

a. First guide in position with the putty bite for transversal pins fixation
b. Transversal pins insertion
c. Hopeless remaining tooth extraction through the first guide
d. Implants bed preparation
e. OT Equator abutments inserted over the implants
f. Temporary cylinders prepared according to the corresponding length inserted over the OT Equator abutments

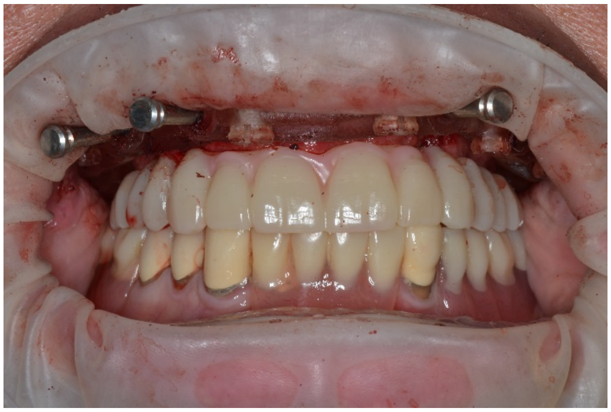

Patient with provisional screwed on the OT Equator abutments.

Follow-Up:

- **One-week follow-up:** The patient presented with the provisional restoration in place, showing satisfactory healing.
- **Panoramic X-ray:** A panoramic X-ray taken one week postoperatively confirmed successful implant placement.
- **Six-month follow-up:** A second panoramic X-ray at six months showed good bone remodeling around the implants.

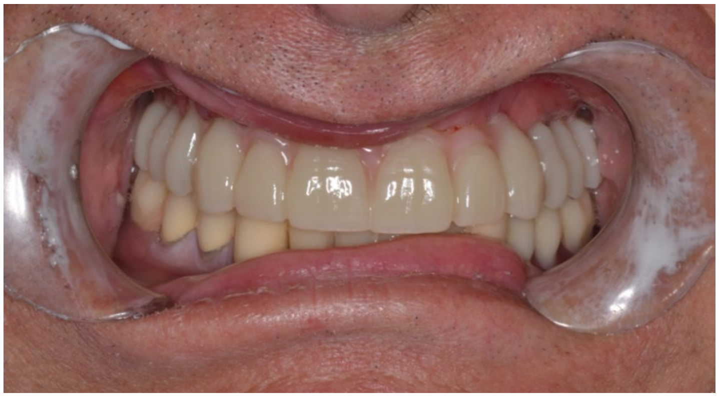

Patient at one-week follow-up

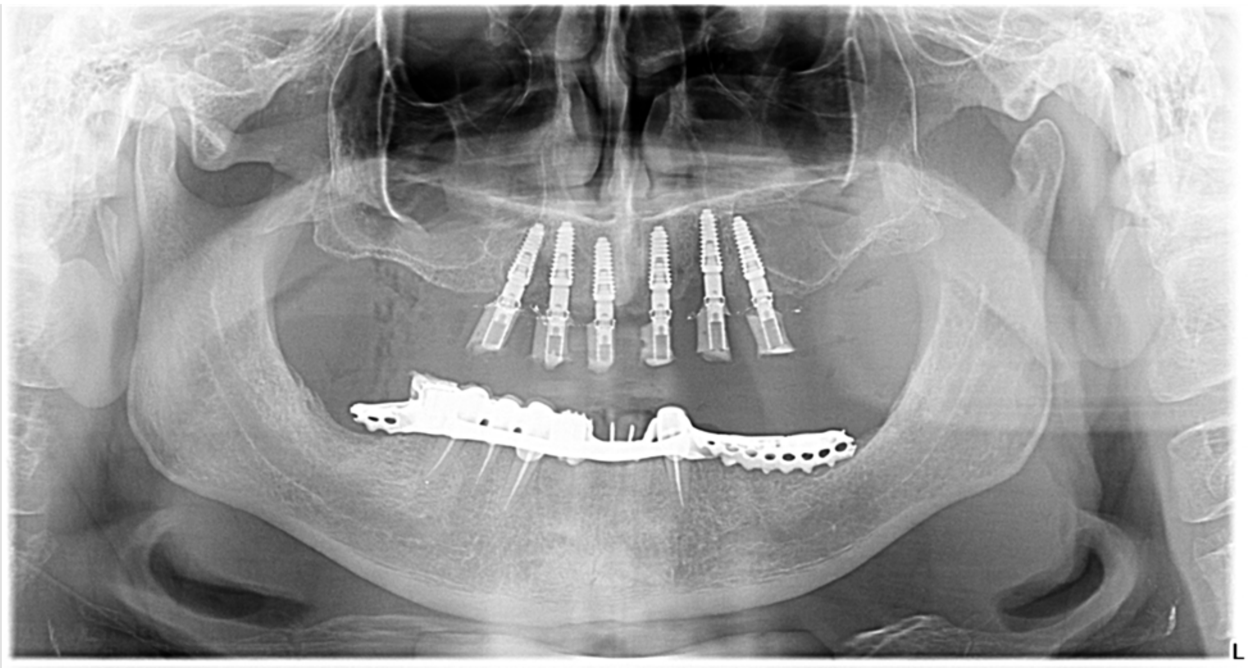

Panoramic x-Ray at one-week follow-up

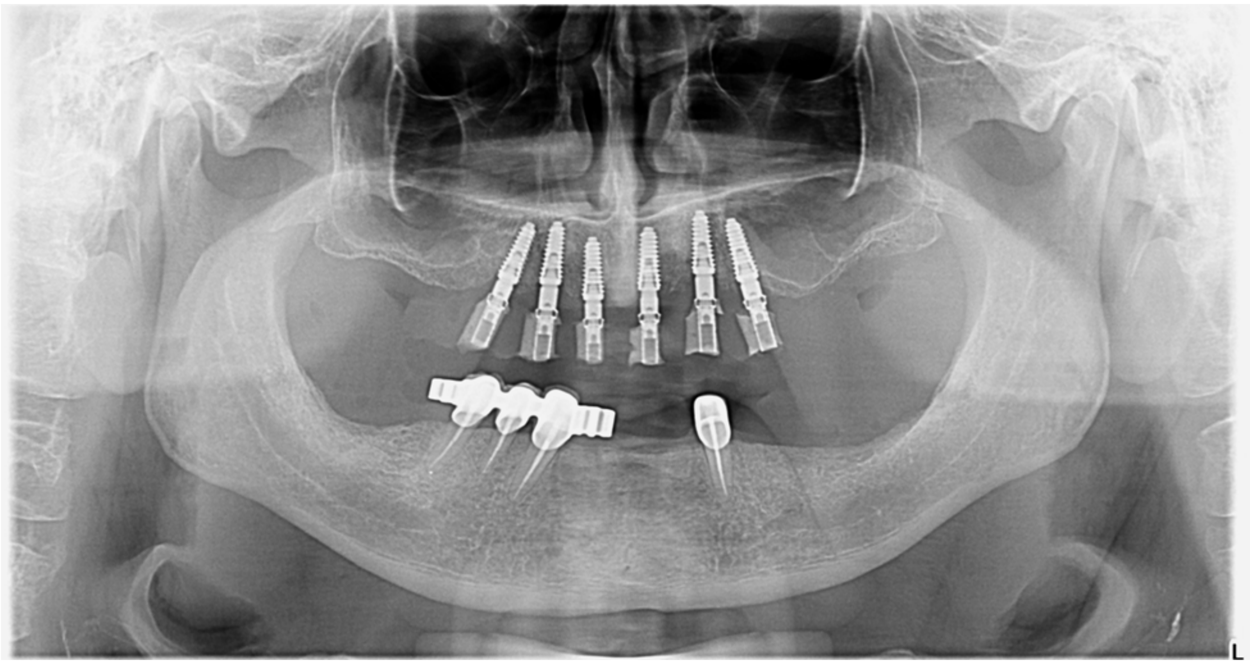

Panormamic X Ray at 6 months follow-up

One-year assessment:

At the one-year follow-up, a CBCT scan was performed to assess the trueness of the implant positioning. Superimposition of the preoperative CBCT with the treatment plan and the one-year postoperative CBCT demonstrated the deviation from the planned implant positions.

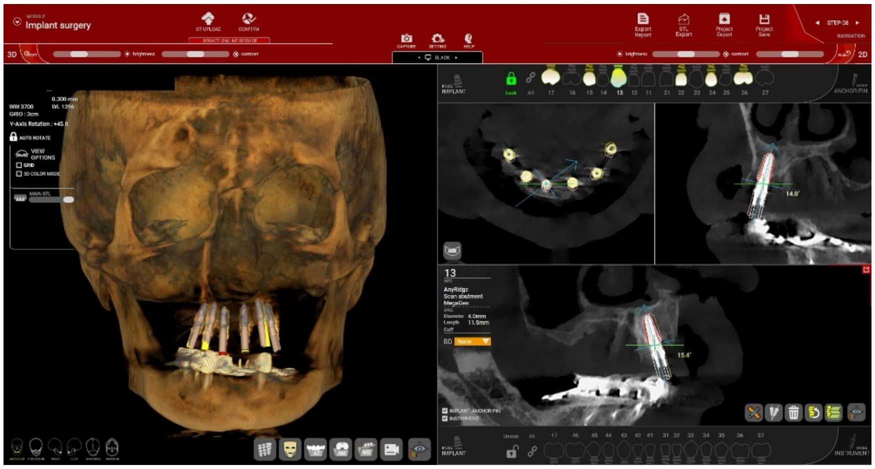

Superimposing pre-op CBCT with the treatment plan with one-year post-op CBCT for trueness assessment.
